# Enhanced Electrochemical Response of Diclofenac at a Fullerene–Carbon Nanofiber Paste Electrode

**DOI:** 10.3390/s19061332

**Published:** 2019-03-17

**Authors:** Sorina Motoc, Florica Manea, Corina Orha, Aniela Pop

**Affiliations:** 1 “Coriolan Dragulescu” Institute of Chemistry, Romanian Academy, Mihai Viteazul 24, Timisoara 300223, Romania; sorinailies@acad-icht.tm.edu.ro; 2 Department of Applied Chemistry and Engineering of Inorganic Compounds and Environment, Politehnica University of Timisoara, P-ta Victoriei no.2, Timisoara 300006, Romania; aniela.pop@upt.ro; 3 National Condensed Matter Department, Institute for Research and Development in Electrochemistry and Condensed Matter, Timisoara, 1 P. Andronescu Street, Timisoara 300254, Romania; orha.corina@gmail.com

**Keywords:** sodium diclofenac, voltammetric/amperometric detection, fullerene–carbon nanofiber paste electrode, real-time water monitoring

## Abstract

The requirements of the Water Framework Directive to monitor diclofenac (DCF) concentration in surface water impose the need to find advanced fast and simple analysis methods. Direct voltammetric/amperometric methods could represent efficient and practical solutions. Fullerene–carbon nanofibers in paraffin oil as a paste electrode (F–CNF) was easily obtained by simple mixing and tested for DCF detection using voltammetric and amperometric techniques. The lowest limit of detection of 0.9 nM was achieved by applying square-wave voltammetry operated under step potential (SP) of 2 mV, modulation amplitude (MA) of 10 mV, and frequency of 25 Hz, and the best sensitivity was achieved by four-level multiple pulsed amperometry (MPA) that allowed in situ reactivation of the F–CNF electrode. The selection of the method must take into account the environmental quality standard (EQS), imposed through the “watchlist” of the Water Framework Directive as 0.1 µg·L^−1^ DCF. A good improvement of the electroanalytical parameters for DCF detection on the F–CNF electrode was achieved by applying the preconcentration step for 30 min before the detection step, which assured about 30 times better sensitivity, recommending its application for the monitoring of trace levels of DCF. The electrochemical behavior of F–CNF as a pseudomicroelectrode array makes it suitable for practical application in the in situ and real-time monitoring of DCF concentrations in water.

## 1. Introduction

The environmental presence of diclofenac, a nonsteroidal anti-inflammatory drug (NSAID), has been found to exhibit toxicological effects on wildlife [1,2], although no direct toxicological effects in human beings have been reported. The environmental quality standard (EQS) for diclofenac that belongs to the “watchlist” of the Water Framework Directive (WFD) was set to 0.1 µg·L^−1^ in surface waters, and its concentrations will be widely monitored through Europe [3].

The monitoring of DCF concentration in aquatic environments necessitates analytical methods, from which several variants of chromatography are often used [4,5]. Also, for the quantitative determination of DCF, electrochemical methods and sensors have been reported [6,7,8,9,10,11,12,13], as electrochemical methods exhibit great potential for environmental monitoring because of their advantages of saving time, fast response, simplicity, low cost, and the avoidance of sample preparation. 

The electrode composition confers the electroanalytical performance of any electrochemical detection method. In general, the direct detection of DCF using a bare electrode is appropriate only for relatively high concentrations of DCF, because the electrochemical response is poor owing to the low electron transfer during electrochemical oxidation on the electrode surface [6,7]. It is well-known that in order to enhance electrochemical performance, the effective strategy is to design a composite consisting of a highly electrocatalytic material and a substrate with good conductivity [12]. The exceptional electrical, chemical, and mechanical properties of carbon nanomaterials mean they have great potential in sensors applications, especially in composite form. The type of nanostructured carbon depends on the synthesis method that is dictated, as well as its price. Examples of nanostructured carbon materials include carbon nanofibers, nanowires, nanotubes, nanoparticles, nanoclusters, and graphene, etc. Also, fullerene (C_60_) belongs to the nanostructured carbon class, and its electrocatalytic properties have been reported for various applications, including electrochemical sensors and detection methods [14,15,16]. The challenge in using nanostructured carbon electrode material for electroanalysis is to obtain inexpensive electrode material characterized by high electrocatalytic performance. The integration of fullerene within a composite electrode should improve the electrocatalytic activity based on its electrochemical behavior as a redox system, due to its remarkable feature of electron-accepting ability [16]. It has been reported that C_60_ can enhance the electron transfer reaction, provides reproducible catalytic responses, and exhibits chemical stability, which makes it an attractive candidate for electroanalytical applications [17,18]. 

Carbon nanofibers (CNF) are considered as one class of the appropriate supportive carbon materials due to the large surface-to-volume ratio and excellent electrical conductivity [19]. Also, they are cheaper in comparison with carbon nanotubes due to the synthesis method [20]. The effect of C_60_ on improving electroanalytical performance in the detection of vinclozolin [21], dopamine [22], and hemoglobin [23] has been reported using carbon nanotube-based supportive materials. In our work, the effect of fullerene (C_60_) on DCF detection using a CNF support is studied. A simple method based on component mixing to obtain an electrode consisting of a paste of fullerene (F) and carbon nanofibers (F–CNF) and investigation of its electrochemical behavior in the presence of sodium diclofenac (DCF) for its electrochemical determination at trace levels in water are described. To our knowledge, no study has been published to date concerning the electroanalytical application of a fullerene-–carbon nanofiber paste electrode. Voltammetric and amperometric techniques, i.e., cyclic voltammetry (CV), differential pulsed voltammetry (DPV), square-wave voltammetry (SWV), chronoamperometry (CA), and multiple pulsed amperometry (MPA), were used to develop enhanced and fast electrochemical methods for DCF determination in aqueous solutions.

## 2. Materials and Methods

The composition of the fullerene–carbon nanofiber paste electrode (F–CNF) was obtained by mixing certain amounts of carbon nanofibers, paraffin oil, and fullerene to reach the ratio of 50 wt. % carbon nanofibers, 25 wt. % fullerene, and 25 wt. % paraffin oil. For comparison, a carbon nanofiber paste electrode (CNF) was similarly obtained with the composition of 75 wt. % carbon nanofibers and 25 wt. % paraffin oil. The mass ratio of fullerene, carbon nanofibers, and paraffin oil of 1:2:1 was chosen to assure the sufficient contribution of fullerene and electrode stability. For comparison, the ratio of 3:1 carbon nanofibers to paraffin oil as the carbon nanofiber paste was used.

The carbon nanofibers (>98% purity), paraffin oil, and fullerene (C_60_, 98% purity) were of analytical standard, provided by Sigma Aldrich (Germany). Fourier transform infrared spectroscopy (FTIR) measurements of F–CNF and CNF in paraffin oil paste were obtained on a Vertex 70 spectrometer from Bruker at room temperature in the wavenumber range of 4000–400 cm^−1^ using transmission technique. The morphological surface characterization of F–CNF in comparison with the simple carbon nanofiber paste electrode (CNF) was studied by a scanning electronic microscope (SEM, Inspect S PANalytical model) coupled with an energy dispersive X-ray analysis detector (EDX).

All the electrochemical measurements were performed using an Autolab potentiostat/galvanostat PGSTAT 302 (Eco Chemie, The Netherlands) controlled with GPES 4.9 software using a three-electrode cell, consisting of a F–CNF paste working electrode, a platinum counter electrode, and a saturated calomel reference (SCE) electrode. The F–CNF paste electrode with disc geometry was obtained by filling a Teflon mold, resulting in an active surface with a diameter of 3 mm. As the supporting electrolyte, 0.1 M sodium sulfate at pH 5 was used. Prior to use, the electrode was electrochemically stabilized through 10 continuous repetitive cyclic voltammograms within the potential ranging between −0.5 and +1.5 V/SCE. Na_2_SO_4_ used was analytical-grade reagent from Merck, and DCF was used as received from Amoli Organics Ltd. All solutions were prepared with doubly distilled and deionised water. 

The electrochemical techniques applied for electrochemical characterization and analytical applications were cyclic voltammetry, differential pulsed voltammetry, square-wave voltammetry, chronoamperometry, and multiple pulsed amperometry. 

## 3. Results and Discussion

### 3.1. Structural and Morphological Characterization

**Figure 1:**
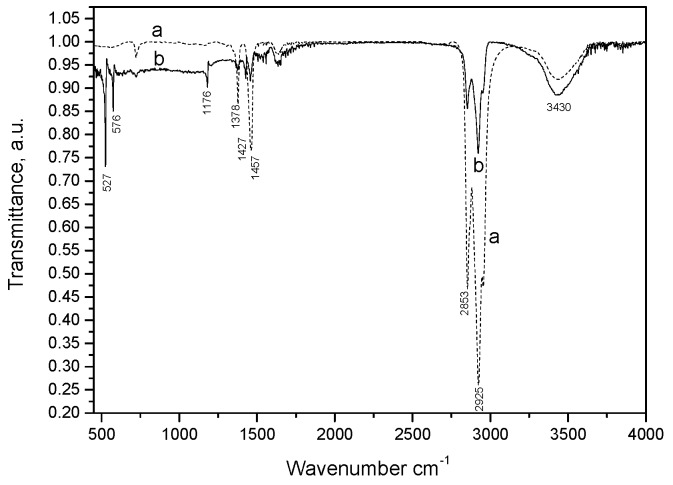
FTIR spectra of carbon nanofiber (CNF)–paraffin oil paste (**a**, dotted line) and C_60_/fullerene (F)–CNF–paraffin oil paste (**b**, solid line).

The molecular structure of the fullerene–CNF paraffin oil paste was characterized by FTIR spectroscopy (Figure 1). In accordance with the literature [24], the peaks recorded at 1427 cm^−1^, 1180 cm^−1^, 576 cm^−1^, and 527 cm^−1^ corresponded to the presence of C_60_. The vibrations seen at 2925 cm^−1^, 2853 cm^−1^, 1457 cm^−1^, 1427cm^−1^, and 1428 cm^−1^ are associated with different aliphatic CH groups (CH and CH_2_ bonds), as reported previously for carbon nanofibers [25]. The broad peak at 3430 cm^−1^ is characteristic of O–H stretching from inter- and intramolecular hydrogen bonds, and the peaks at 1652 cm^−1^ and 1457 cm^−1^ are characteristic of phenolic resins [26].

**Figure 2:**
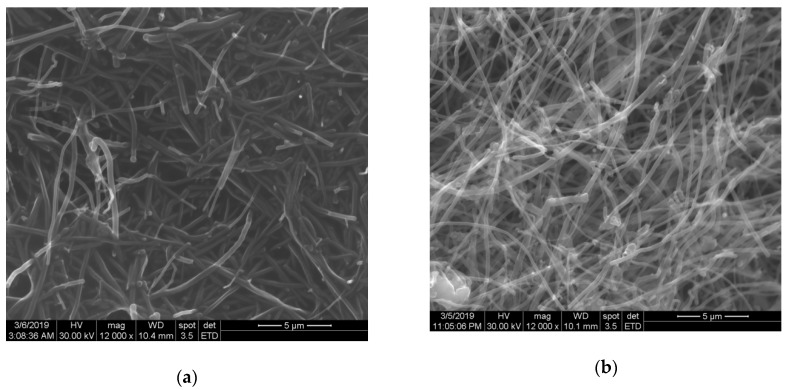
SEM images of (**a**) CNF–paraffin oil paste and (**b**) F–CNF–paraffin oil paste.

The electrode paste composition morphology was studied through SEM and the results are presented in Figure 2. A good distribution of both carbon nanofibers and fullerene in oil paraffin was assured, and a randomized arrangement of both carbon nanofibers and fullerene resulted. 

### 3.2. Cyclic Voltammetry

Cyclic voltammetry using the classical potassium ferri/ferrocyanide redox system was used for the determination of the electroactive area of the fullerene–CNF–paraffin oil paste electrode. Cyclic voltammetry (CV) of the supporting electrolyte consisting of 4 mM K_3_[Fe(CN)_6_] in 1 M KNO_3_ was recorded at different scan rates (results not shown here), and the diffusion coefficient was determined as 10.86 × 10^−6^ cm^2^·s^−1^ according to the Randles–Sevcik Equation (1):

(1) $$I_{p}=2.69\times 10^{5}AD^{1/2}n^{3/2}v^{1/2}C$$

where *A* represents the area of the electrode (cm^2^), *n* is the number of electrons participating in the reaction (and is equal to 1), *D* is the diffusion coefficient of the molecule in solution, *C* is the concentration of the probe molecule in the solution and is 4 mM, and *v* is the scan rate (V·s^−1^); the linear dependence between peak current and the square root of the scan rate was achieved. Taking into account the theoretical diffusion coefficient value of 6.7 × 10^−6^ cm^2^·s^−1^ found in the literature data [27], the value of the electroactive electrode area was determined to be 0.249 cm^2^ versus the value of the electrode geometric area of 0.196 cm^2^.

**Figure 3:**
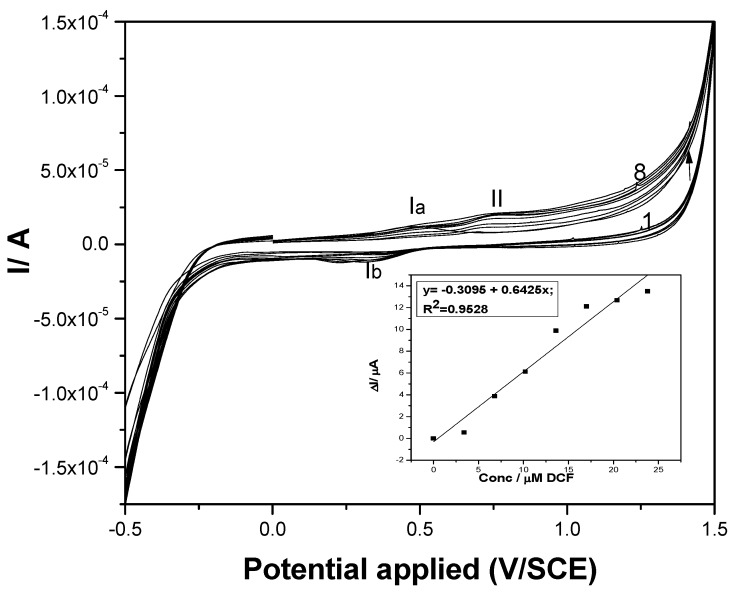
Cyclic voltammograms recorded at the F–CNF paste electrode in 0.1 M Na_2_SO_4_ supporting electrolyte (curve 1) and in the presence of various DCF concentrations: curves 2–8: 1–7 mg·L^−1^ DCF. Inset: Calibration plots of the currents versus DCF concentrations at potential value of +0.75 V/SCE.

The electrochemical behavior of DCF on both paste electrodes was investigated by cyclic voltammetry (CV) in a supporting electrolyte of 0.1 M Na_2_SO_4_. No peak corresponding to the DCF oxidation appeared for the carbon nanofiber paste electrode without fullerene content (results not shown here). This should be explained by the absence of an electrocatalytic effect of CNF towards DCF electrooxidation, or by a large background current recorded on the simple carbon nanofiber paste electrode increasing at each usage and thus overlapping the electrochemical response for DCF electrooxidation. The latter would also give information about the instability of the electrode composition. CV series recorded on the F–CNF paste electrode at various DCF concentrations are presented in Figure 3, and an anodic peak corresponding to DCF oxidation is evidenced at the potential value of about +0.75 V/SCE (peak II). It must be noticed that the CV shape showed the presence of anodic and corresponding cathodic peaks (Ia and Ib) characteristics in the carbon redox system [28]. The first stage in the overall oxidation of DCF is given by the DCF sorption onto the carbon surface, evidenced by the diminution of the anodic peak of carbon oxidation, followed by the second stage of the DCF oxidation process. It has been reported that the electrochemical oxidation of diclofenac involves a one-electron electrochemical-chemical (EC) mechanism followed by a chemical reaction in which 2,6 dichloroaniline and 2-(2-hydroxyprop-2-phenyl) acid acetic are formed [10]. A linear dependence between the useful anodic peak current and DCF concentration is noticeable in the inset of Figure 3. 

**Figure 4:**
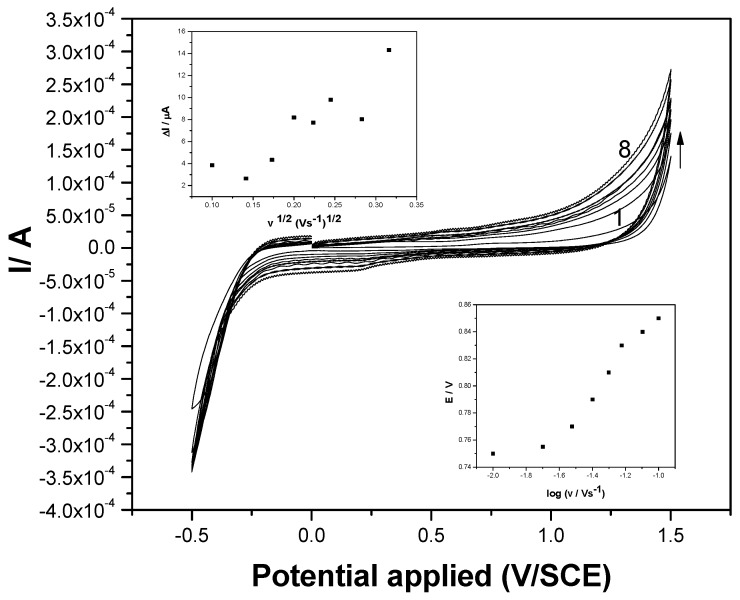
Cyclic voltammograms recorded at the F–CNF paste electrode in 5 mg·L^−1^ DCF and 0.1 M Na_2_SO_4_ supporting electrolyte at various scan rates: (1) 10, (2) 20, (3) 30, (4) 40, (5) 50, (6) 75, (7) 100, and (8) 200 m·Vs^−1^. Insets: upper: dependence of anodic peak current vs. square root of the scan rate; lower: dependence of peak potential vs. logarithm of the scan rate.

Some mechanistic aspects related to the electrooxidation of DCF on the F–CNF paste electrode can be discussed through the study of the scan rate influence. CVs recorded in the presence of 5 mg·L^−1^ DCF and 0.1 M Na_2_SO_4_ supporting electrolyte at the scan rates ranging from 0.01 to 0.2 V·s^−1^ are presented in Figure 4. From these CVs, it can be noticed that the dependence between the anodic peak current and the square root of the scan rate is not linear (see inset of Figure 4), which denotes a nonlinear diffusion-controlled oxidation process. This should be explained by the fact that the F–CNF paste electrode can work as a pseudomicroelectrode array, which is characterized by spherical and nonlinear diffusion patterns that are specific to macroelectrodes. Also, surface-controlled or complex processes should influence the linearity of the dependence between the anodic peak current and the square root of the scan rate. A complex process involving fullerene’s availability to act as a multiple electron acceptor [16] in DCF oxidation should be considered to explain nonlinear diffusion. The irreversible characteristic of the overall oxidation process is evidenced by the lack of the cathodic peak corresponding to the anodic DCF oxidation, and also through the dependence of the oxidation potential value and the logarithm of the scan rate. 

### 3.3. Analytical Applications

In order to develop the electroanalytical methods for the determination of DCF, two approaches were considered. The first one considered differential pulsed and square-wave voltammetries (DPV and SWV) for enhancing the sensitivity and the lowest limit of detection (LOD) of DCF. The second considered the chronoamperometry and multiple pulsed amperometry (CA and MPA), being the simplest and fastest electrochemical methods for DCF determination.

#### 3.3.1. DPV and SWV

**Figure 5:**
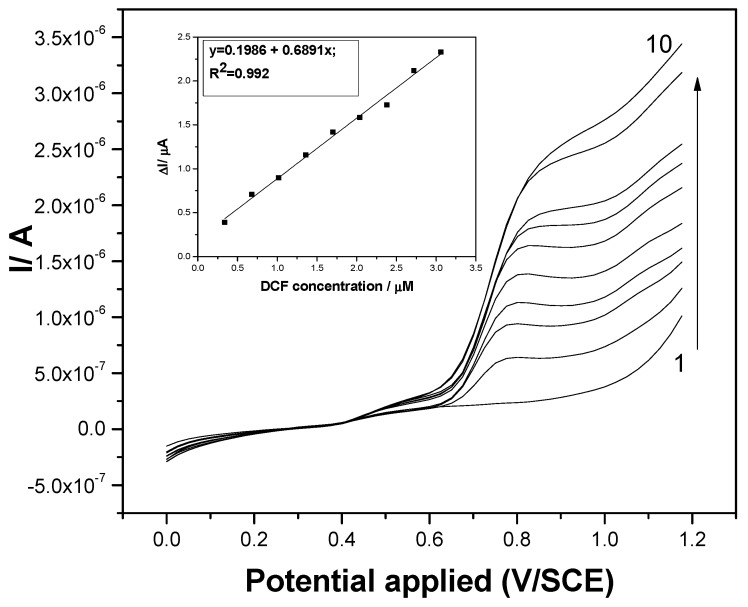
Differential pulsed voltammograms recorded at the F–CNF paste electrode in 0.1 M Na_2_SO_4_ supporting electrolyte (curve 1) and in the presence of various DCF concentrations: curves 2–10: 0.1–0.9 mg·L^−1^ DCF; step potential (SP) 25 mV; modulation amplitude (MA) 100 mV; potential range: 0 to +1.2 V/SCE. Inset: Calibration plots of the currents recorded at E = +0.75 V/SCE versus DCF concentrations.

Both voltammetric techniques were applied under optimized operating conditions applied for the electrochemical determination of DCF on a boron-doped diamond (BDD) electrode as reported in our previous work [7]. DPV technique was applied at an SP of 25 mV, an MA of 100 mV, and at the scan rate of 0.05 V·s^−1^ and differential pulsed voltammograms are shown in Figure 5. A good linearity between the anodic peak current recorded at +0.75 V/SCE and DCF concentration was reached (see inset of Figure 5). It must be mentioned that more than tenfold higher sensitivity was achieved using the F–CNF paste electrode in comparison with the BDD electrode operated under the same conditions [7]. A slight enhancement in sensitivity was achieved using DPV under these operating conditions. However, about six times lower LOD and respective limit of quantification (LOQ) were obtained, which proved the DPV to be superior in comparison with CV.

**Figure 6:**
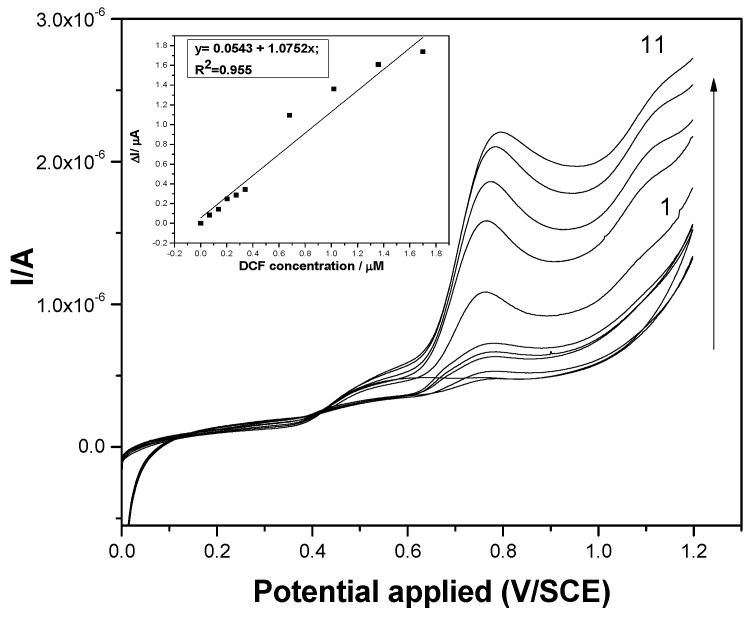
Square-wave voltammograms recorded at the F–CNF paste electrode in 0.1 M Na_2_SO_4_ supporting electrolyte (curve 1) and in the presence of various DCF concentrations: curves 2–6: 0.02–0.1 mg·L^−1^ DCF and curves 7–11: 0.2–0.6 mg·L^−1^ DCF; step potential (SP) 2 mV; modulation amplitude (MA) 10 mV; potential range: 0 to +1.2 V/SCE. Inset: Calibration plots of the currents recorded at E = +0.75 V/SCE versus DCF concentrations.

Also, the SWV technique was tested under similarly optimized operating conditions reported by our group for the BDD electrode [7], and the results are presented in Figure 6. A larger DCF concentration range was detected using SWV operated under SP of 2 mV, MA of 10 mV, and frequency of 25 Hz, and a good linearity was reached, as can be seen in the inset of Figure 6. About two times better electroanalytical parameters were reached in comparison with the results of DPV.

**Figure 7:**
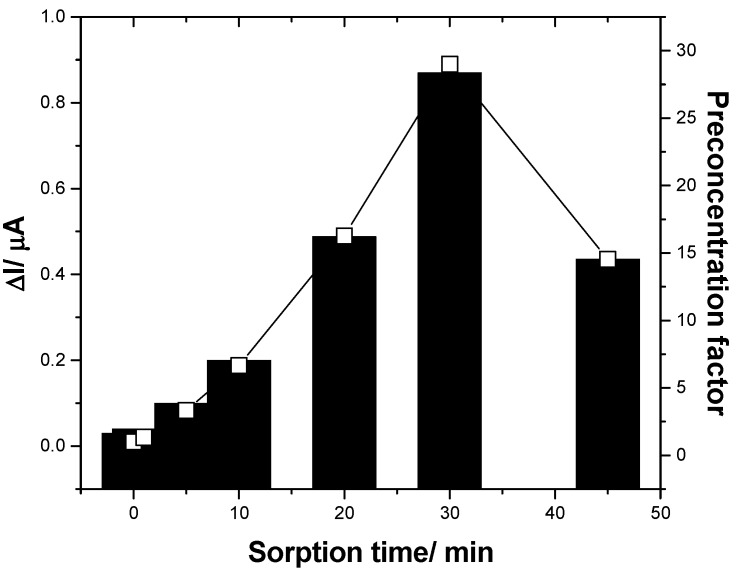
Useful signals reached by square-wave voltammetry (SWV) recorded in the presence of 0.005 mg·L^−1^ DCF containing 0.1 M Na_2_SO_4_ supporting electrolyte at F–CNF paste electrodes, as a function of the sorption time in the preconcentration step prior to detection.

Regarding the sorption properties of fullerene for DCF, this inconvenience should be exploited in a positive way to improve the electroanalytical parameters of both sensitivity and, especially, LOD by inclusion of the preconcentration step before the detection step applied for low DCF concentrations. In the preconcentration step, the F–CNF paste electrode is immersed in supporting electrolyte containing low DCF concentrations at the open circuit potential for a certain time to assure its sorption onto the electrode surface. A maximum preconcentration factor of about 28 was found at 30 min (see Figure 7). A longer sorption time led to diminution of the preconcentration factor, probably due to the fouling effect starting to manifest. It is clear that this preconcentration–detection scheme necessities a longer time for DCF determination, but this is nevertheless useful for trace concentration levels of DCF.

#### 3.3.2. CA and MPA

**Figure 8:**
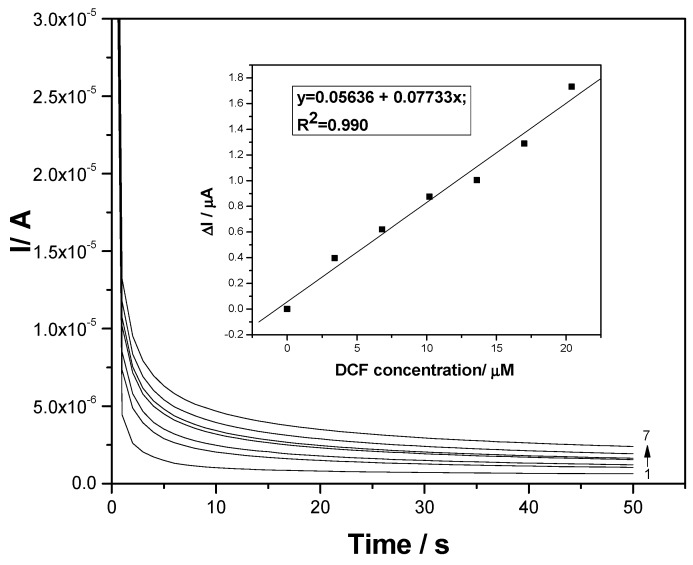
Chronoamperograms (CAs) recorded for a single level of the detection potential of +1 V/SCE at the F–CNF paste electrode in 0.1 M Na_2_SO_4_ supporting electrolyte (curve 1) and in the presence of various DCF concentrations: curves 2–7: 1–6 mg·L^−1^ DCF. Inset: Calibration plots of the currents versus DCF concentrations.

Considering the simple, easy, and fast attributes of CA and MPA, various amperometric schemes were tested to obtain very good electroanalytical parameters. Conventional CA tested at a single level of the potential value of +1 V/SCE, which is higher than a value recommended by the CV through the DCF oxidation peak (+0.75 V/SCE), allowed us to reach CAs recorded at various DCF concentrations presented in Figure 8. Lower sensitivity was reached by CA, probably due to the fouling effect of the electrode surface.

**Figure 9:**
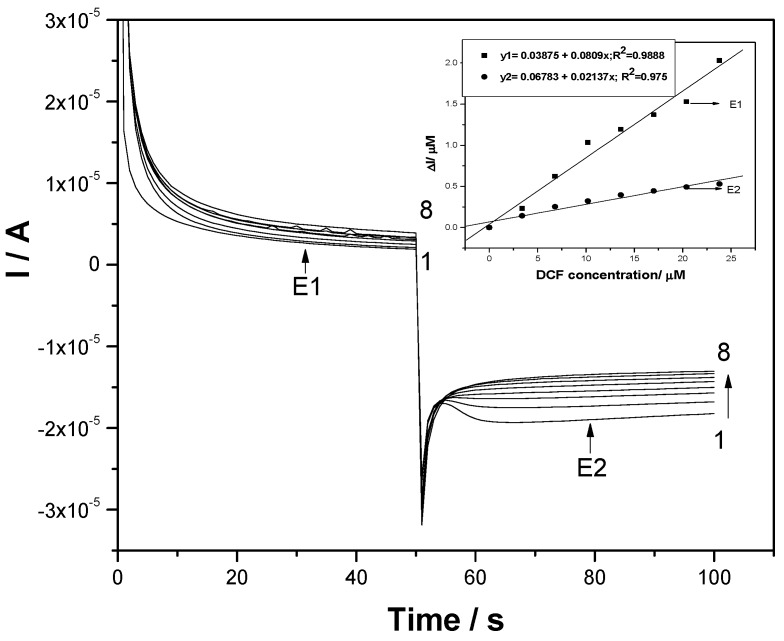
Chronoamperograms (CAs) recorded for two levels of detection potential, namely +1 V/SCE and activation potential of −0.3 V/SCE (E2), at the F–CNF paste electrode in 0.1 M Na_2_SO_4_ supporting electrolyte (curve 1) and in the presence of various DCF concentrations: curves 2−8: 1−7 mg L^−1^ DCF. Inset: Calibration plots of the currents versus DCF concentrations at both potential values (E1 and E2).

To assure the cathodic activation of the F–CNF paste electrode, CAs operating at the two potentials of +1 V/SCE and −0.3 V/SCE were applied, and the results are shown in Figure 9. The cathodic potential belongs to the hydrogen evolution potential range, being selected in accord with CV. It can be noticed that a decreasing cathodic current occurs with DCF concentration increasing, which suggested that this simple procedure did not allow the activation of the F–CNF paste electrode at this medium cathodic potential value and no enhanced response was reached under these operating conditions (see inset of Figure 9). 

**Figure 10:**
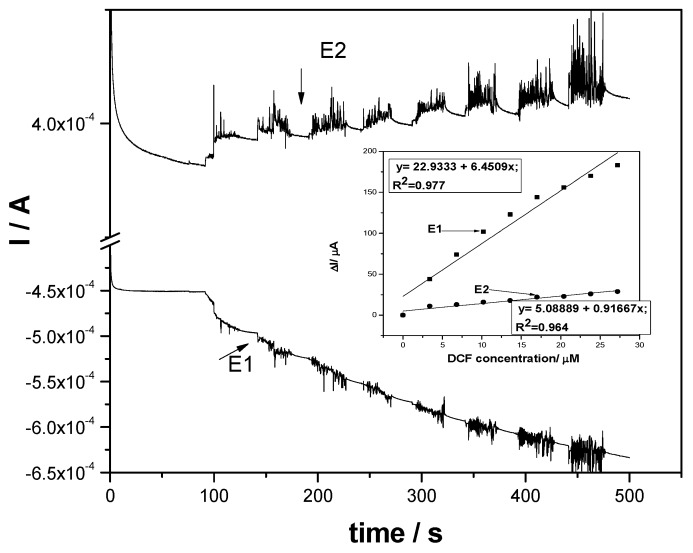
Multiple pulsed amperograms (MPAs) recorded for two levels of the potential pulses of +1 V/SCE for 0.05 s (E1) and activation potential of −0.3 V/SCE for 0.05 s (E2) at the F–CNF paste electrode in 0.1 M Na_2_SO_4_ supporting electrolyte (curve 1) and in the presence of various DCF concentrations: 1–7 mg·L^−1^ DCF. Inset: Calibration plots of the currents versus DCF concentrations at both potential pulse values (E1 and E2).

Under these circumstances of involving sorption processes in the anodic oxidation and detection of DCF on the F–CNF paste electrode, which was reflected also in the low sensitivity when using chronoamperometry with one and two levels of potential, multiple pulsed amperometry (MPA) was tested under several strategies in order to improve the electronalaytical parameters for DCF detection. It is well-known that pulsed amperometric detection involves in situ cleaning and reactivation of the electrode surface during the electrodetection process [29]. The responses of MPA corresponded to each potential pulse applied for a short duration, combining the anodic and cathodic polarization and avoiding the fouling effect on the electrode surface. The first variant of MPA applied consisted of the application of similar potentials with two levels of CA. By continuously applying the same potential values for the short duration of 0.05 s per pulse, the amperograms recorded at the F–CNF paste electrode at −0.3 V/SCE and +1 V/SCE are shown in Figure 10. Enhanced sensitivities were reached for MPA in comparison with CA for both anodic and cathodic potential pulses (see inset of Figure 10). The short durations of pulse application impeded the sorption and fouling effect manifestation, which led to the better efficiency of DCF detection. Ten times higher sensitivity at the potential value of +1 V/SCE was achieved; also, the cathodic current increased linearly with DCF, increasing at the potential value of −0.3 V/SCE, and a commendable sensitivity was reached, being markedly higher than that recorded at the anodic part. The cathodic part assured the very good in situ reactivation of the electrode surface. 

**Figure 11:**
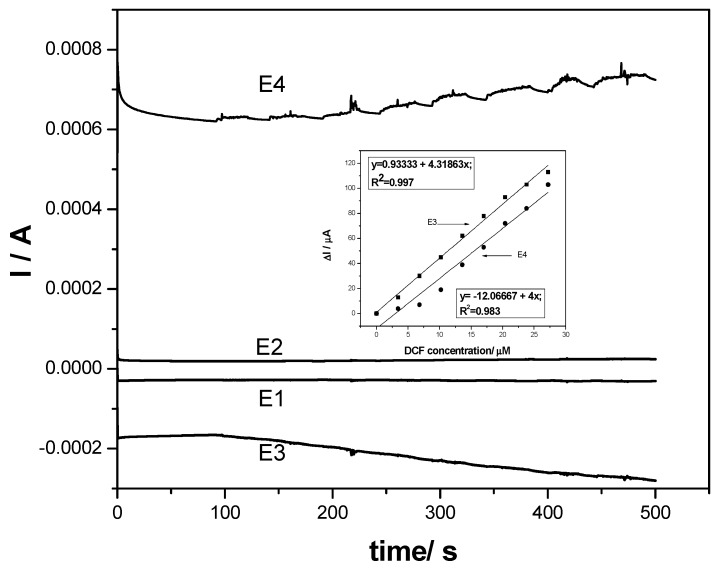
Multiple pulsed amperograms (MPAs) recorded for four levels of the potential pulses of +0.3 V/SCE for 0.1 s (E1), +0.5 V/SCE for 0.1 s (E2), −0.3 V/SCE for 0.1 s (E3), and +1 V/SCE for 0.05 s (E4) at the F–CNF paste electrode in 0.1 M Na_2_SO_4_ supporting electrolyte and in the presence of various DCF concentrations: 1–7 mg·L^–1^ DCF. Inset: Calibration plots of the currents versus DCF concentrations at both E3 and E4 potential pulses.

Another strategy was considered for MPA, applying it in according with the CV shape as the reference, involving the redox system of fullerene that should act as an electrocatalyst in DCF oxidation and detection, and the amperograms are shown in Figure 11.

The pulses were applied continuously using the following scheme:

1. +0.3 V/SCE for a duration of 100 ms, where fullerene is in the reduced form;
2. +0.5 V/SCE for a duration of 100 ms, where reduced fullerene is oxidized;
−0.3 V/SCE for a duration of 100 ms, where H_2_ evolution occurs alongside other reduction processes;
4. +1 V/SCE for a duration of 50 ms, considering the detection potential that corresponded to DCF oxidation.

It is obvious that the better electroanalytical parameters were achieved using MPA under the operating conditions presented above. The currents recorded at E1 and E2 potential values did not vary linearly with DCF concentration due to fullerene-related surface processes occurring, which significantly influenced the DCF oxidation process and, implicitly, the detection sensitivity due to the fact that the reduced fullerene can act as an efficient electron mediator for DCF oxidation, leading to the considerable enhancement of the analytical sensitivity [14]. About four times better sensitivity at the detection potential of +1 V/SCE was achieved by integration of both E1 and E2 potential pulses within the MPA-based detection strategy in comparison with two-level MPA-based detection strategy. All electroanalytical parameters determined for each electrochemical technique and detection scheme are summarized in Table 1.

It can be noticed that the lowest limits of detection and quantification were reached when applying SWV, while the best sensitivities were achieved by the MPA technique. In comparison with other electrodes reported in the literature for voltammetric/amperometric detection of DCF [6,7,8,12], the F–CNF paste electrode exhibited enhanced electroanalytical performance regarding both sensitivity and the lowest limit of detection.

#### 3.3.3. Analysis of DCF in Spiked Tap Water

**Figure 12:**
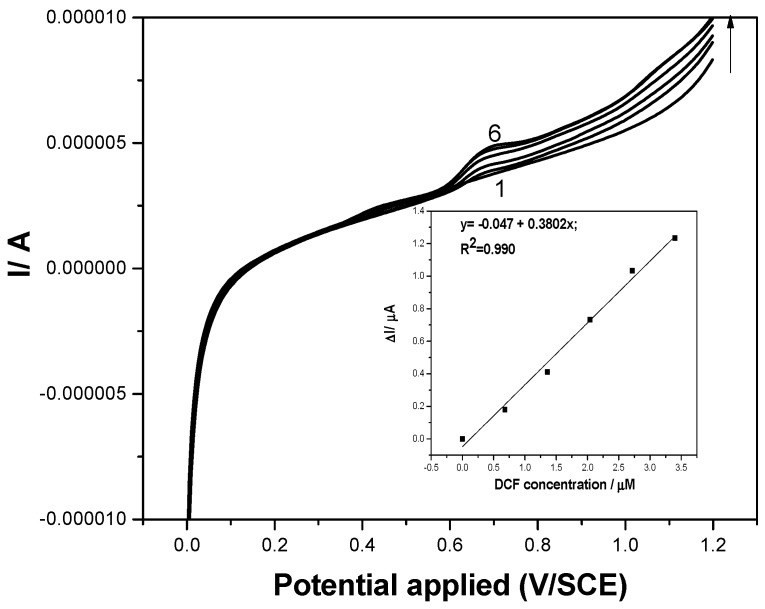
Square-wave voltammograms recorded on the F–CNF paste electrode in tap water without supporting electrolyte (curve 1) and in the presence of various DCF concentrations: curves 2–6: 0.1–0.5 mg·L^−1^ DCF; step potential (SP) 2 mV; modulation amplitude (MA) 10 mV; potential range: 0 to +1.2 V/SCE. Inset: Calibration plots of the currents recorded at E = +0.75 V/SCE versus DCF concentrations.

The C_60_/fullerene-modified carbon nanofiber paste (F–CNF) electrode was directly used to determine the presence of DCF in tap water, envisaging its detection in a real water matrix without deliberately adding any supporting electrolyte. Not every type of electrode is able to detect an analyte without supporting electrolyte; only one that can act as an array/ensemble of microelectrodes. From the effect of the scan rate study presented above, a pseudomicroelectrode array behavior was concluded to occur, which justified the further testing of the electrode to detect DCF in tap water. A different calibration plot was determined for SWV in application with tap water for DCF concentrations ranging from 1 to 5 mg·L^−1^. A smaller sensitivity of 0.38 μA·µM^−1^ was achieved in tap water (Figure 12) in comparison with the sensitivity of 1 μA·µM^−1^ reached in 0.1 M Na_2_SO_4_ supporting electrolyte (Figure 6), which concluded that calibration is required in a real water matrix.

Based on this calibration plot, the practical analytical application of the proposed SWV method was further established by determining DCF concentrations in tap water without any preliminary treatment. A recovery test was performed by analyzing three parallel tap water samples, which were spiked with 1 and 5 mg·L^−1^ DCF. The recovery test was run directly in tap water without supporting electrolyte. The recovery values higher than 95% and the relative standard deviation (RSD) values less than 5% for both concentrations indicated good recovery and reproducibility of the results and the great potential of the F–CNF paste electrode to be used for in situ and real-time water quality monitoring.

Repeatability of the sensor was evaluated by comparing the results of the determination of a solution containing 5 mg·L^−1^ DCF over three days. The relative standard deviation of less than 4% demonstrated an appropriate repeatability of the proposed sensor. The electrode was tested for a time period of two months, and a 99% electrochemical response was found at the end of this period for 5 mg·L^−1^ DCF, which indicated a good stability and life time.

## 4. Conclusions

C_60_/fullerene–carbon nanofibers in paraffin oil in a weight ratio of 1:2:1, comprising a F–CNF paste electrode, exhibited good dispersion of both carbon fillers and chemical stability in both Na_2_SO_4_ supporting electrolyte and tap water. Its electrochemical behavior in the presence of sodium diclofenac (DCF) studied by cyclic voltammetry made it appropriate to be tested in some variants of voltammetric/amperometric-based analytical applications. In comparison with the performance of a boron-doped diamond electrode for DCF detection [7], the F–CNF electrode showed enhanced voltammetric/amperometric response, due to the electrocatalytic effect of the fullerene towards the anodic oxidation of DCF. The lowest limit of detection of 0.9 nM was achieved by applying square-wave voltammetry operated under SP of 2 mV, MA of 10 mV, and frequency of 25 Hz, which is appropriate for detecting DCF concentrations, according to environmental quality standard (EQS) imposed through the “watchlist” of the Water Framework Directive. Also, a good improvement of the electroanalytical parameters for DCF detection on the F–CNF electrode was achieved by applying a preconcentration step for 30 min before the detection step, which assured about 30 times better sensitivity. Simple, fast, and good electrochemical response for DCF detection was achieved by four-level multiple pulsed amperometry (MPA) that allowed in situ reactivation of the F–CNF paste electrode. However, this method can be selected for DCF concentrations that exceed the EQS in water samples, or for application with pharmaceutical formulations. The electrochemical peculiarities of the F–CNF electrode, as a pseudomicroelectrode array, make it appropriate for in situ and online analytical applications for DCF monitoring in real surface water.

## Figures and Tables

**Table 1:** Electroanalytical parameters for DCF detection on the F–CNF paste electrode.

Technique	Conditions, E/V vs. SCE	Sensitivity μA·µM^−1^	Correlation Coefficient (R^2^)	LOD ^a^ (µM)	LQ ^b^ (µM)	RSD ^c^ (%)
CV	+0.75	0.642	0.952	0.0568	0.1893	0.1531
DPV	+0.77	0.689	0.992	0.0102	0.0341	1.0097
SWV	+0.75	1.076	0.955	0.0009	0.0029	0.1028
CA	+1 V	0.077	0.990	0.0905	0.3019	0.3733
CA	+1 V	0.080	0.988	1.2788	4.2628	1.8678
	−0.3 V	0.021	0.975	4.4203	14.7345	1.7280
MPA	−0.3 V−0.05 s	6.450	0.977	6.1520	20.5068	2.8758
	+1 V−0.05 s	0.916	0.964	14.9974	49.9915	1.1871
MPA	+0.3 V−0.1 s	- ^d^	-	-	-	-
	+0.5 V−0.1 s	-	-	-	-	-
	−0.3 V−0.1 s	4.318	0.997	3.1324	10.4413	2.6473
	+1 V−0.05 s	4.000	0.983	1.8874	6.2915	0.4043

^a,b^ The lowest limit of detection and the lowest limit of quantification, respectively, determined in accordance with the literature [30]; ^c^ For three replicates; ^d^ - means not determined.
